# The Effect of Sintering Temperature on the Phase Composition, Microstructure, and Mechanical Properties of Yttria-Stabilized Zirconia

**DOI:** 10.3390/ma15082707

**Published:** 2022-04-07

**Authors:** Volodymyr Kulyk, Zoia Duriagina, Bogdan Vasyliv, Valentyna Vavrukh, Taras Kovbasiuk, Pavlo Lyutyy, Volodymyr Vira

**Affiliations:** 1 Department of Materials Science and Engineering, Lviv Polytechnic National University, 12 S. Bandera Str., 79013 Lviv, Ukraine; zduriagina@ukr.net (Z.D.); vavrukh.valentyna@gmail.com (V.V.); taras.m.kovbasiuk@lpnu.ua (T.K.); pavlo_lyutyy@ukr.net (P.L.); 2 Department of Materials Engineering, The John Paul II Catholic University of Lublin, 14 Racławickie Al., 20-950 Lublin, Poland; 3 Department of Hydrogen Technologies and Alternative Energy Materials, Karpenko Physico-Mechanical Institute, 5 Naukova Str., 79060 Lviv, Ukraine; 4 Department of Strength of Materials and Structural Mechanics, Lviv Polytechnic National University, 12 S. Bandera Str., 79013 Lviv, Ukraine; viravolodymyr@gmail.com

**Keywords:** YSZ ceramics, microstructure, microhardness, fracture toughness, fracture micromechanism

## Abstract

It is known that the yttria-stabilized zirconia (YSZ) material has superior thermal, mechanical, and electrical properties. This material is used for manufacturing products and components of air heaters, hydrogen reformers, cracking furnaces, fired heaters, etc. This work is aimed at searching for the optimal sintering mode of YSZ ceramics that provides a high crack growth resistance. Beam specimens of ZrO_2_ ceramics doped with 6, 7, and 8 mol% Y_2_O_3_ (hereinafter: 6YSZ, 7YSZ, and 8YSZ) were prepared using a conventional sintering technique. Four sintering temperatures (1450 °C, 1500 °C, 1550 °C, and 1600 °C) were used for the 6YSZ series and two sintering temperatures (1550 °C and 1600 °C) were used for the 7YSZ and 8YSZ series. The series of sintered specimens were ground and polished to reach a good surface quality. Several mechanical tests of the materials were performed, namely, the microhardness test, fracture toughness test by the indentation method, and single-edge notch beam (SENB) test under three-point bending. Based on XRD analysis, the phase balance (percentages of tetragonal, cubic, and monoclinic ZrO_2_ phases) of each composition was substantiated. The morphology of the fracture surfaces of specimens after both the fracture toughness tests was studied in relation to the mechanical behavior of the specimens and the microstructure of corresponding materials. SEM-EDX analysis was used for microstructural characterization. It was found that both the yttria percentage and sintering temperature affect the mechanical behavior of the ceramics. Optimal chemical composition and sintering temperature were determined for the studied series of ceramics. The maximum transformation toughening effect was revealed for ZrO_2_-6 mol% Y_2_O_3_ ceramics during indentation. However, in the case of a SENB test, the maximum transformation toughening effect in the crack tip vicinity was found in ZrO_2_-7 mol% Y_2_O_3_ ceramics. The conditions for obtaining YSZ ceramics with high fracture toughness are discussed.

## 1. Introduction

Modern techniques of designing novel structural materials intended for applications in various high-temperature structural components are currently being developed. These techniques include fine-grained microstructure formation due to the optimization of material manufacturing modes allowing materials to reach excellent high-temperature strength and crack growth resistance as well as thermal stability [1,2,3,4,5,6,7,8,9].

In particular, the superalloys used in state-of-the-art gas turbines operate close to their upper limits of temperature capability and thermal stability because of the demand for increasing gas turbine efficiency and higher firing temperature. Therefore, the application of thermal barrier coatings (TBCs) to the combustor and high-pressure turbine blades has become urgent [10]. Yttria-stabilized zirconia (YSZ) based TBCs have already exhibited excellent thermal characteristics. However, the 4% volume difference between m-ZrO_2_ (monoclinic) and t-ZrO_2_ (tetragonal) phases [11] is a cause of coating failure under thermal cycling conditions. Therefore, stabilization of the t-ZrO_2_ phase is a key issue in TBC development. The addition of rare earth metal oxides was found to be very effective in solving this problem. The authors [12] conducted a high-temperature XRD analysis to investigate the phase transformation and changes in lattice parameters of various phases in the basic YSZ ceramics and a co-doped one with trivalent oxides Sc_2_O_3_ and Yb_2_O_3_. They found a tendency towards the thermal stabilization of the microstructure for the co-doped specimens when the test temperature was above the critical value. The lattice parameters for all specimens increased with temperature in XRD analysis. The lattice parameters for 7 mol% YSZ are larger than that for the two trivalent rare earth oxides co-doped with YSZ under the same temperature.

The authors of another work [13] investigated the phase stability of RE_2_O_3_ (RE = La, Nd, Gd, Yb) and Yb_2_O_3_ co-doped Y_2_O_3_-stabilized ZrO_2_ ceramics and concluded that the phase stability of the compounds increased with the decrease in the RE^3+^ size. They showed that this phenomenon could be attributed to the reduced driving force for partitioning of the t’ phase.

The authors [14] studied the effect of doping/co-doping on the high-temperature phase compositions of YSZ ceramics using XRD analysis from room temperature to 1100 °C. YSZ specimens without doping and CeO_2_ and Nb_2_O_5_ co-doped YSZ specimens were investigated. It was found that the monoclinic phase dominated in Nb_2_O_5_ co-doped YSZ at temperatures below 600 °C, while the cubic/tetragonal phase dominated in both the YSZ and CeO_2_ co-doped YSZ in a temperature range from room temperature to 1100 °C.

Obtaining rare earth-doped ZrO_2_ with high sintering resistance and good phase stability is an urgent task to be solved by materials scientists who work in the field of high-temperature protective coatings. The authors [15] studied the effect of dopant species (La_2_O_3_, Nd_2_O_3_, Gd_2_O_3_, and Y_2_O_3_) on the sintering resistance and phase stability of zirconia-based ceramics. When ZrO_2_ was doped with the ions with larger radii (La^3+^, Nd^3+^, and Gd^3+^), it exhibited improved sintering resistance at reduced tetragonal phase stability. It was found based on molecular dynamics simulation results that rare earth ions with larger ionic radii were disposed to segregation at grain boundaries, which can more effectively reduce the grain boundary energy in the materials under consideration. The proposed mode involving doping ZrO_2_ with NdO_1.5_ (1 mol%) and YbO_1.5_ (6 mol%) is considered promising for manufacturing ZrO_2_-based ceramics for refractory and thermal barrier materials.

In the work [16], the possibilities of application of a photoluminescence method for damage diagnostics of TBCs have been studied. Two types of coatings were investigated: (1) coating based on undoped YSZ powder; (2) coating based on YSZ powder doped with 2 mol% Eu^3+^. The coatings were heat-treated at 1100 °C for 100, 300, and 800 h. The authors used SEM analysis for studying the morphology of the interface between the topcoat (TBC) and the bond coat and the indentation method [17] for estimating the apparent interfacial toughness. It was found that the partial substitution of Y^3+^ ions by a low amount of Eu^3+^ ions did not cause discernible changes in the microstructural components or any lowering in the interfacial toughness of the YSZ coatings. Additionally, no diffusion of Eu^3+^ into the thermally grown oxide layer was found. It was concluded that the partial substitution of Y^3+^ ions by Eu^3+^ ions is promising since no deterioration of the properties of TBCs was revealed.

The authors [18] studied the high-temperature properties of ZrO_2_—25 wt.% CeO_2_—2.5 wt.% Y_2_O_3_ (CYSZ) for applications in TBCs. It was proposed to substitute micro-CYSZ with nano-CYSZ. The nanopowder was synthesized by the sol-gel method followed by calcination for 3 h at 1000 °C and subsequently underwent a heat treatment at 1300 °C for 50 h. By using XRD and Raman analyses, the authors confirmed the formation of the non-transformable (t´) ZrO_2_ phase as well as the stability of this phase after heat treatment. Based on the properties of nano-CYSZ, they suggested the material as promising for advanced TBCs in aero-engine and power generation applications.

In the work [19], the effects of powder particle morphology and size on microstructure and phase composition of Y_2_O_3_-ZrO_2_ polycrystals were studied. The authors used dilatometry measurements to investigate the powder’s compact behavior during sintering. The SEM and EBSD studies allowed for identifying symmetry between the observed grains. Hardness, fracture toughness, and mechanical strength measurements were also performed. Two populations of grains essentially differing in their sizes were found. Surprisingly, the EDS line scan of the bigger grains displayed substantially higher yttrium content than in the much smaller grains surrounding them. The X-ray diffraction of the material revealed the presence of 46.6% t-ZrO_2_ phase, 15.6% c-ZrO_2_ phase, and 37.8% m-ZrO_2_ phase. Using EBSD analysis, the authors tried to attribute corresponding symmetry to the grains observed in the specimen microstructure. They stated that the preferential matter transport from nanometric Y_2_O_3_-ZrO_2_ particles towards sub-micrometer particles led to the transformation of the latter to form a higher-symmetry part of the system. They also suggested two mechanisms related to this phenomenon. One resulted from the yttrium concentration gradient, but it would lead to the chemical homogenization of the system. The second mechanism was related to the high curvature of the contact points between small and larger grains. This leads to the matter diffusion of smaller grains toward larger ones. These grains, coming initially from sub-micrometric monoclinic particles, become sufficiently rich in yttrium to develop t-ZrO_2_ phase and c-ZrO_2_ phase symmetry. Simultaneously, nanometric particles initially rich in yttrium transfer to the part of the microstructure featuring monoclinic symmetry. The described phenomenon does not allow for the chemical homogenization of the system.

The authors [20] studied the microstructure evolution of two ZrO_2_-SiO_2_ nanocrystalline glass-ceramics (NCGCs) in relation to thermal treatment modes. NCGCs were composed of monoclinic (m) and tetragonal (t) ZrO_2_ nanocrystallites and an amorphous SiO_2_ compound. During thermal treatment, both m-ZrO_2_ and t-ZrO_2_ nanocrystallites were metastable. The metastability of m-ZrO_2_ and t-ZrO_2_ nanocrystallites was explained using a size-driven phase transformation approach. It was shown that the percentage of m-ZrO_2_ in the undoped ZrO_2_-SiO_2_ NCGC increased due to thermal treatment at 850 °C for 5 h and decreased due to thermal treatment at a temperature above 950 °C. A rapid phase transformation of t-ZrO_2_ nanocrystallites was reached due to thermal treatment at 1250 °C for 5 h followed by cooling, with the formation of 88.6 vol% m-ZrO_2_. In contrast, the yttria additive was the reason for improved t-ZrO_2_ phase stability to a temperature of 1250 °C, since the percentage of m-ZrO_2_ in the yttria-doped ZrO_2_-SiO_2_ NCGC continuously decreased with an increase in a temperature of thermal treatment up to 1250 °C. The sizes of both m-ZrO_2_ and t-ZrO_2_ nanocrystallites increased with an increase in the temperature of thermal treatment for both the NCGCs.

In the work [21], a comparative study on densification and microstructural evolution of 8 mol% YSZ sintered ceramics reinforced with CeO_2_ particles (10, 12, and 14 wt.% CeO_2_) has been performed. The specimens were fabricated via both microwave and conventional sintering methods. In both cases, the sintering temperature was 1400 °C, and the holding time was 20 min and 5 h for the microwave and conventional methods, respectively. The materials were characterized in terms of densification, microstructure, and mechanical behavior. For both methods, the sintered densities of 8YSZ specimens increased with the addition of CeO_2_ amount. In these conditions, no destabilization of the 8YSZ cubic crystal structure was found. It was revealed that the grain size of the 8YSZ specimens decreased with the addition of CeO_2_. Respectively, Vickers hardness of the ceramics increased with increasing CeO_2_ amount. All the mentioned effects were found to be more pronounced in microwave sintered specimens compared to those obtained by the conventional method.

The authors of the works [22,23] studied the phase changes in plasma sprayed YSZ coatings during annealing. It was found using neutron scattering and XRD studies that the t-ZrO_2_ phase decomposed into the m-ZrO_2_ phase and c-ZrO_2_ phase while the yttria amount in the t-ZrO_2_ phase decreased. Using XRD analysis, the authors of the works [24,25] revealed that in the plasma-sprayed and EB-PVD coatings under study, the transformation of the t-ZrO_2_ phase into a yttria-depleted t-ZrO_2_ phase and a c-ZrO_2_ phase or t-ZrO_2_ phase with high yttria content occurred in a temperature range of 1300–1400 °C. Transmission electron microscopy (TEM) along with XRD analysis were used in the work [26] for studying the phase transformations in EB-PVD coatings heat-treated in a temperature range of 1100–1500 °C. It was found that the t-ZrO_2_ phase decomposed into an yttria-depleted t-ZrO_2_ phase and both the t-ZrO_2_ phase and c-ZrO_2_ phases with high Y_2_O_3_ content. It was also revealed that the domain boundaries having a cubic-like structure contain a quite large number of yttrium ions [27,28]. Such knowledge allows for increasing the energy efficiency of high-temperature fuel cells by optimizing the operating modes and microstructure of ceramic electrodes [29,30,31,32]. This urgent task is caused by the deployment of renewables to meet global climate objectives [33,34].

It is critically important to manufacture materials resistive to the aggressive operating environment [35,36,37,38] to avoid microstructure degradation [39,40,41,42]. Therefore, along with strength and tribology tests of materials as the most popular methods for diagnosing their load-bearing capacity, the indentation test, known as the simplest mechanical method [39,43], is widely used. This method is more microstructurally sensitive as compared to the above-mentioned ones and allows for estimating the crack growth resistance of materials [40,44]. Fracture toughness tests employing various specimen shapes and loading schemes are also quite microstructurally sensitive [40,44,45]. Therefore, the application of microhardness and crack growth resistance test methods for diagnostics of the microstructure stability of YSZ ceramics is promising in terms of searching for the optimal sintering and treatment modes.

This work is aimed at evaluating the effect of sintering temperature on the phase composition, the size and morphology of the crystallites, and the mechanical properties of YSZ ceramics stabilized by the various amount of yttria.

## 2. Experimental Procedures

In this work, yttria-stabilized zirconia (YSZ) ceramics sintered from commercial starting powders have been studied. The powders were produced at the Vol’nogorskii Mining and Smelting Plant, Vol’nogorsk, Ukraine. Initial particle sizes of the starting powders were as follows: 100–150 nm (ZrO_2_ powder) and 10–30 nm (Y_2_O_3_ powder). A series of beam specimens of YSZ ceramics stabilized with 6, 7, and 8 mol% Y_2_O_3_ (hereinafter: 6YSZ, 7YSZ, and 8YSZ) approximately 4.2 × 4.2 × 50 mm in size were sintered in a furnace for 2 h. Argon was used as an inert sintering atmosphere. Eight variants of material were obtained using four sintering temperatures (1450 °C, 1500 °C, 1550 °C, and 1600 °C) for the 6YSZ series and two sintering temperatures (1550 °C and 1600 °C) for the 7YSZ and 8YSZ series (Table 1). For marking each variant, corresponding chemical composition and sintering temperature were indicated, e.g., 7YSZ-1550. After sintering, the side surfaces of specimens were polished using a grinding and polishing machine for metallographic preparation to reach the required surface quality and avoid phase transformations.

Microhardness of the material variants was measured using a NOVOTEST TC-MKB1 microhardness tester (Novotest, Novomoskovsk, Ukraine). We used the following set of indentation loads: 0.49 N, 0.98 N, 1.96 N, 2.94 N, 4.91 N, and 9.81 N. At least 10 indentations for each level of the indentation load were made to determine the microhardness of each material variant.

The relevant standards [46,47] regulate the microhardness measurement conditions. Vickers microhardness {in GPa} is calculated by the formula [47]:

(1) $$H=0.0018544\left(\frac{P}{d^{2}}\right)$$

where *P* is the indentation load {N} and *d* is the average length of the diagonals of the indentation imprint {mm}.

An optical microscope Neophot-21 (Zeiss, Oberkochen, Germany) was used for estimating the imprint and crack geometry.

Along with microhardness, the fracture toughness of material was estimated by to calculating the critical stress intensity factor (SIF), *K*_Ic_. This characteristic made it possible to characterize the propensity of a material to brittle fracture due to the nucleation and propagation of cracks [48,49,50]. There exists a wide range of methods for estimating the fracture toughness of materials under Vickers pyramid indentation [42,51,52]. In these works, the formulas for calculating the *K*_Ic_ values contain both physical and mechanical parameters, as well as empirical coefficients. Due to the comparison of the *K*_Ic_ values calculated by these formulas with those obtained by conventional methods of fracture mechanics, we recently concluded [53] that the following formula presented by the authors of the work [51] best fits the characterization of the ZrO_2_-Y_2_O_3_ ceramics:

(2) $$K_{\mathrm{Ic}}=0.016{\left(\frac{E}{H}\right)}^{1/2}\left(\frac{P}{c^{3/2}}\right)$$

where *E* is Young’s modulus {GPa}, *H* is microhardness {GPa}, *P* is the indentation load {N}, and *c* is the radial crack length {m}.

Therefore, we used this formula to estimate the fracture toughness of the materials under study.

For comparison, we used in this work a single-edge notch beam (SENB) test [54,55,56] to estimate the fracture toughness of the material. An edge notch less than 0.1 mm in width was machined in a beam specimen. The three-point bending SENB tests were carried out on the MTS Criterion E43.104 test machine (MTS Systems Corporation, Eden Prairie, MN, USA) at 20 °C in air. The distance between the supporting rollers of the loading unit was 14 mm. For calculating the critical SIF *K*_Ic_ of material, we used corresponding formulas [54,55,56]. The average *K*_Ic_ value was computed for each set of five specimens of the investigated material variants.

The material microstructure and morphology of the fracture surfaces of tested specimens were investigated using a Carl Zeiss EVO-40XVP scanning electron microscope (SEM) (Zeiss, Oberkochen, Germany). The chemical homogeneity of materials was evaluated with an energy-dispersive X-ray (EDX) microanalysis using an INCA Energy 350 system (Oxford Instruments, Abingdon, UK). A DRON-4.07M diffractometer (Bourevestnik, St. Petersburg, Russia) was used to perform X-ray diffraction (XRD) studies of as-sintered specimens. All procedures including indexing, refinement of the profile and structural parameters, as well as calculations/evaluation of the phase weight fractions were performed using the WinCSD program package (WinCSD, https://www.wincsd.eu/, accessed on 20 March 2022). The ZrO_2_ phase marking and reference codes were as follows: t–tetragonal (COD ID 2300612), m–monoclinic (COD ID 1528984), and c–cubic (COD ID 2101234).

## 3. Results and Discussion

### 3.1. The XRD Analysis of YSZ Ceramics

**Figure 1:**
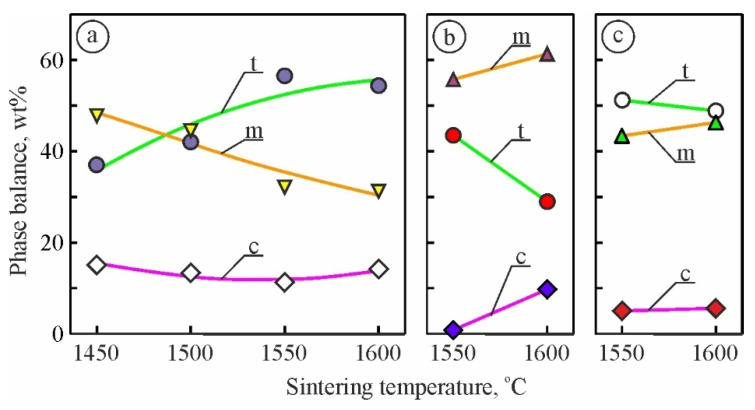
Changes in the zirconia phase balance of the investigated materials of variants (**a**) 6YSZ-1450, 6YSZ-1500, 6YSZ-1550, 6YSZ-1600, (**b**) 7YSZ-1550, 7YSZ-1600, and (**c**) 8YSZ-1550, 8YSZ-1600 depending on the sintering temperature (see Table 1). Phase marking: t—tetragonal, m—monoclinic, c—cubic.

The obtained XRD patterns of the materials under study (Table 1) exhibit, in general, the phase balance for 6YSZ, 7YSZ, and 8YSZ ceramics (Figure 1). We revealed ambiguous changes in the phase balance with changes in the sintering temperature from 1450 °C to 1600 °C for 6YSZ ceramics. A maximum percentage of t-ZrO_2_ (over 56 wt.%) along with a decrease in m-ZrO_2_ and c-ZrO_2_ weight fractions (to 32 wt.% and 11 wt.%, respectively) was revealed for this material sintered at a temperature of 1550 °C.

Thus, in 6YSZ ceramics, the sintering temperature of 1550 °C allows for providing a relatively high percentage of the t-ZrO_2_ phase, while the m-ZrO_2_ phase weight fraction is decreased compared to variants 6YSZ-1450 and 6YSZ-1500. Similarly, the c-ZrO_2_ phase weight fraction reaches its minimum for variant 6YSZ-1550. In 7YSZ ceramics at the sintering temperature of 1550 °C, a relatively high percentage of the m-ZrO_2_ phase (over 55 wt.%) was obtained, while the t-ZrO_2_ phase weight fraction (about 43 wt.%) obtained was lower than for both variants 6YSZ-1550 and 8YSZ-1550.

**Figure 2:**
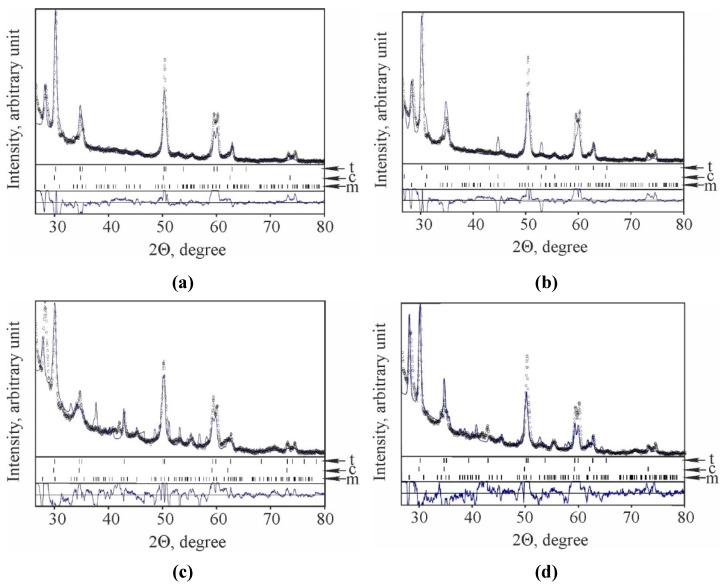
XRD patterns of the investigated materials of variants (**a**) 6YSZ-1550, (**b**) 6YSZ-1600, (**c**) 7YSZ-1600, and (**d**) 8YSZ-1600 (see Table 1). Phase marking: t—tetragonal, m—monoclinic, c—cubic.

The XRD patterns of the selected material variants (Figure 2, variants 6YSZ-1550, 6YSZ-1600, 7YSZ-1600, and 8YSZ-1600) show in detail the above-mentioned peculiarities of the phase balance of the studied compositions.

The XRD pattern of 6YSZ ceramics of variant 6YSZ-1550 contains peaks of the t-ZrO_2_, c-ZrO_2_, and m-ZrO_2_ phases (Figure 2a). The peaks of the c-ZrO_2_ phase are quite low, corresponding to about 11 wt% in this phase (Figure 1a). In contrast, the t-ZrO_2_ phase peaks are high corresponding to about 56 wt% in this phase. The XRD pattern of variant 6YSZ-1600 (Figure 2b) contains higher peaks of the c-ZrO_2_ phase and lower peaks of the t-ZrO_2_ phase as compared to the XRD pattern of variant 6YSZ-1550 (Figure 2a), whereas peaks of the m-ZrO_2_ phase are of the same height for both variants.

The material of variant 7YSZ-1600 is characterized by much higher peaks of the m-ZrO_2_ phase and lower peaks of the t-ZrO_2_ phase (Figure 2c) as compared to variant 6YSZ-1600 sintered in the same mode (Figure 2b).

The XRD pattern of variant 8YSZ-1600 (Figure 2d) exhibits a behavior differing from those of both 6YSZ-1600 and 7YSZ-1600 variants (Figure 2b and Figure 2c, respectively). This variant is intermediate in terms of peak height of both the t-ZrO_2_ and m-ZrO_2_ phases, whereas the lowest peaks of the c-ZrO_2_ phase are observed for it.

Therefore, we can describe the general tendencies of changes in 6YSZ, 7YSZ, and 8YSZ ceramics phase compositions as follows: (i) with increasing sintering temperature, the content of the tetragonal phase increases when the percentage of the stabilizing Y_2_O_3_ additive is quite low (6YSZ ceramics); (ii) the sintering temperature of 1550 °C is critical in 6YSZ, 7YSZ, and 8YSZ ceramics since the content of the tetragonal phase decreases and content of the monoclinic phase increases with a further increase in sintering temperature; (iii) the amount of cubic phase is quite low, especially in 7YSZ and 8YSZ ceramics, so the cubic phase is the balance; (iv) the maximum m-ZrO_2_ phase percentage was found in variant 7YSZ-1600 as a result of the decrease in the t-ZrO_2_ phase weight fraction and increase in the c-ZrO_2_ phase weight fraction.

In general, the phase balance in the ceramics under study reflects the competing effect of two factors; namely, the sintering temperature of the ceramics and the content of the stabilizing Y_2_O_3_ additive. Therefore, to achieve a relatively high content of metastable tetragonal phase, all the ceramics investigated (6YSZ, 7YSZ, and 8YSZ) should be sintered at a temperature of 1550 °C. Under these conditions, a minimum amount of cubic phase is formed.

### 3.2. Mechanical Properties of YSZ Ceramics and the Relations to Their Microstructure

In the work [53], a dependence of the microhardness of yttria-stabilized zirconia (ZrO_2_–8 mol% Y_2_O_3_) on the indentation load, known as the indentation size effect [57], was revealed. For this material, the average values of microhardness decreased with increasing indentation load from 0.49 N to 9.81 N. Additionally, a tendency was observed with the yield of microhardness values on the plateau at indentation loads in a range of 4.91 N to 9.81 N. It was concluded that the values of fracture toughness and microhardness obtained in this range of indentation loads are invariant.

**Figure 3:**
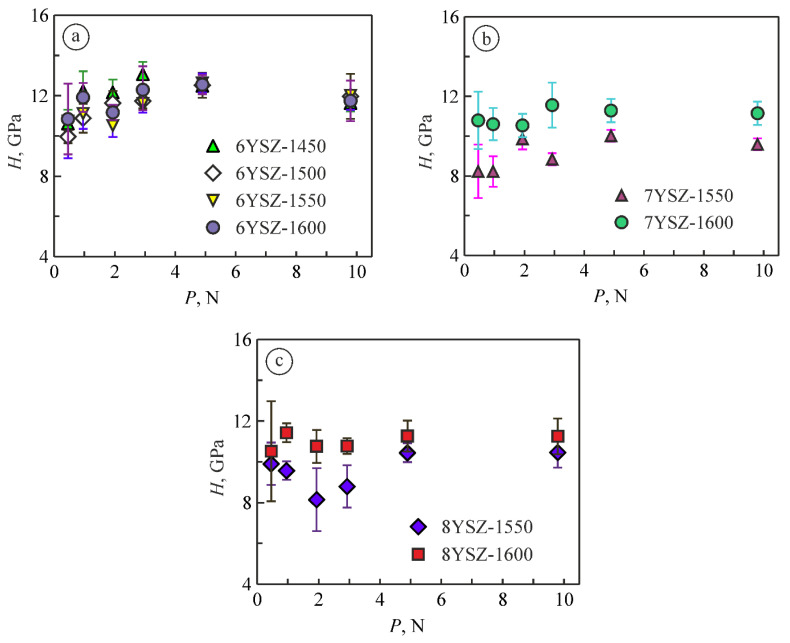
Dependences of microhardness on the indentation load for (**a**) 6YSZ, (**b**) 7YSZ, and (**c**) 8YSZ ceramics sintered at a temperature of 1450 °C (variant 6YSZ-1450), 1500 °C (variant 6YSZ-1500), 1550 °C (variants 6YSZ-1550, 7YSZ-1550, and 8YSZ-1550), and 1600 °C (variants 6YSZ-1600, 7YSZ-1600, and 8YSZ-1600). The variant numbers (see Table 1) correspond to the symbol numbers.

In our work, the dependences of microhardness on the indentation loads of 0.49 N, 0.98 N, 1.96 N, 2.94 N, 4.91 N, and 9.81 N for the material variants 1–8 were obtained (Figure 3). It was found that the material variants 7YSZ-1600 and 8YSZ-1600 are characterized by decreasing the average values of microhardness with increasing indentation load. The phase balance of these ceramics is characterized by the maximum percentages of the monoclinic phase. However, for other material variants, we can observe the opposite tendency (Figure 3).

In general, the yield of average microhardness values on the plateau at higher indentation loads was found for all the material variants.

**Figure 4:**
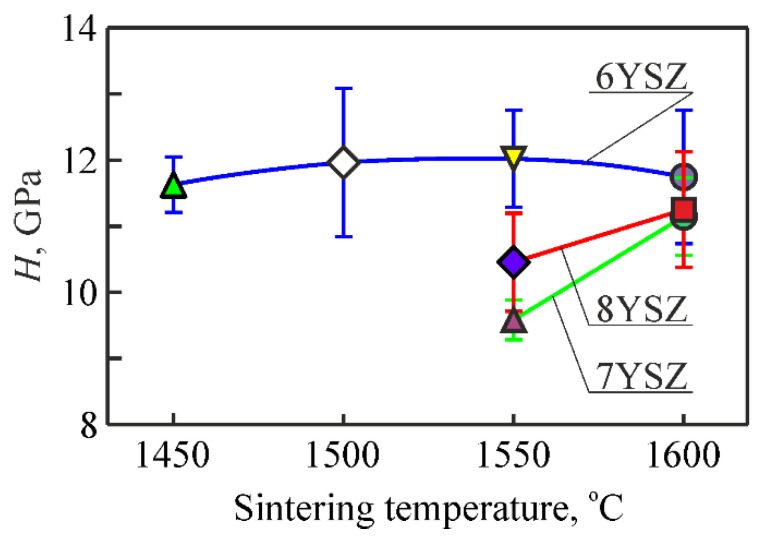
Changes in microhardness of 6YSZ, 7YSZ, and 8YSZ ceramics depending on the sintering temperature (see Table 1). The microhardness was measured under the indentation load of 9.81 N.

**Figure 5:**
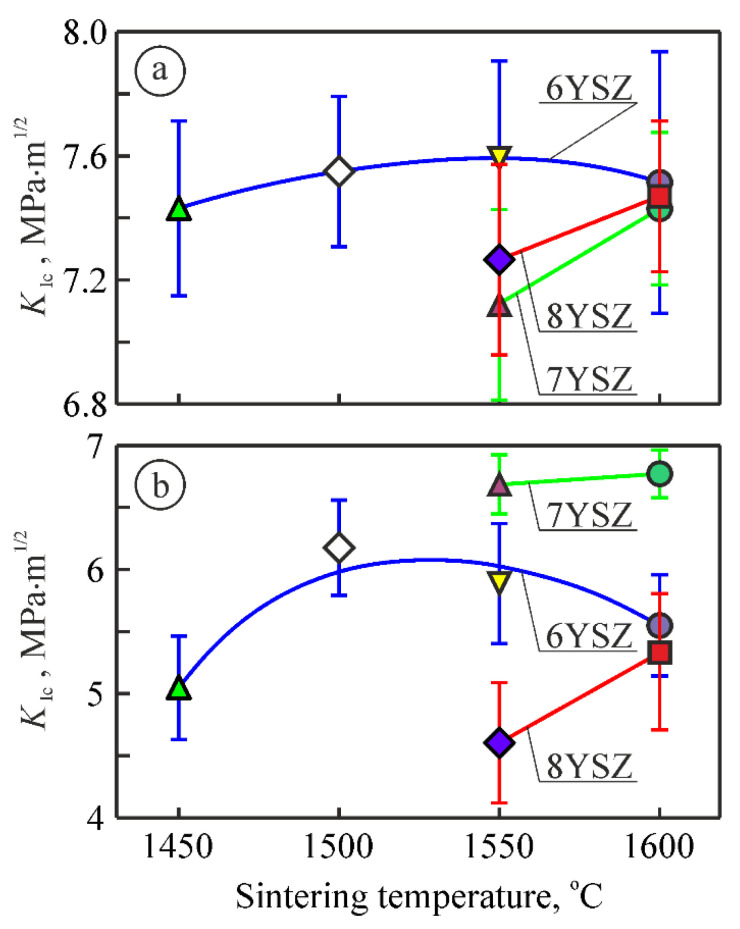
Changes in fracture toughness of 6YSZ, 7YSZ, and 8YSZ ceramics depending on the sintering temperature (see Table 1): (**a**) Vickers indentation method under the indentation load of 9.81 N; (**b**) SENB method under three-point bending.

The invariant values of the material microhardness obtained under the indentation load of 9.81 N were taken to construct a graph for studying the evolution of changes in microhardness of the studied ceramic variants with a change in the sintering temperature from 1450 °C to 1600 °C (Figure 4). It was revealed that, generally, the increase of sintering temperature from 1450 °C to 1500 °C leads to the improvement of mechanical properties of 6YSZ ceramics. In particular, an increase in microhardness (by 5–6%, Figure 4) was observed for this material and the same tendency in fracture toughness (by 3–4%, Figure 5a) was found. The levels of these characteristics remain unchanged while increasing the sintering temperature up to 1550 °C.

The increase in sintering temperature up to 1600 °C leads to intensive grain growth in YSZ ceramics. This, in turn, leads to the suppression of retention of the metastable tetragonal zirconia [58] in the case when the average grain size of the t-ZrO_2_ phase is larger than the admissible one. It seems that the last is about 1 μm for ceramics of this type. According to [58], the microhardness of m-ZrO_2_ is lower than t-ZrO_2_. During indentation, the t-m transition occurs with the formation of m-ZrO_2_ which causes the lowering of microhardness.

In contrast to 6YSZ ceramics, a substantial increase in the m-ZrO_2_ weight fraction for 7YSZ and 8YSZ ceramics was revealed while increasing the sintering temperature from 1550 °C to 1600 °C (Figure 1). This, in turn, leads to a decrease in the t-ZrO_2_ or c-ZrO_2_ weight fractions in these ceramics.

A common result of the two above-mentioned processes, namely, suppression of the t-m transformation of ZrO_2_ with the sintering temperature increase and the stress-induced formation of m-ZrO_2_, is displayed in both Figure 4 and Figure 5a. For the case of the sintering temperature of 1550 °C, the differences between the average values of both microhardness (Figure 4) and fracture toughness (by the Vickers indentation method, Figure 5a) for 7YSZ and 8YSZ ceramics are observed. The latter has an advantage over the previous one both in terms of microhardness and fracture toughness. Probably, a high percentage of m-ZrO_2_ in 7YSZ ceramics (Figure 1b) causes the lowering of microhardness, whereas a slight effect of the t-m transformation occurs at a comparatively low percentage of t-ZrO_2_. In contrast, the microhardness of 8YSZ ceramics is higher because of a lower percentage of m-ZrO_2_ (Figure 1c). On the other hand, since the t-ZrO_2_ percentage is higher in 8YSZ ceramics (Figure 1c), the t-m transformation has a significant effect on microhardness by lowering it and, oppositely, causes an increase in fracture toughness of the material (Figure 4 and Figure 5a, respectively).

On the contrary, another pattern of calculated critical SIF *K*_Ic_ values using the SENB method was obtained (Figure 5b). Significantly higher fracture toughness of 7YSZ ceramics as compared to 6YSZ and 8YSZ (by 15% and 46%, respectively) was found. This ambiguous behavior of the material is evidence showing that the sintering temperature of about 1550 °C is critical in the microstructure formation process. Such an ambiguity during the estimation of fracture toughness of material by two different methods showed that the t-m transition that occurred in the crack tip vicinity of a notched beam specimen is more pronounced than in the case of the Vickers pyramid indentation. In contrast, no appropriate conditions were available for enhancing the fracture toughness of 8YSZ ceramics, and the reason for that was a lower percentage of m-ZrO_2_ and, especially, c-ZrO_2_.

For material sintered at 1600 °C, the t-m transformation dynamics are less pronounced. In this case, mainly t-ZrO_2_ by its strength, without the contribution from the t-m transformation, provides the achieved level of crack growth resistance (Figure 5a) which is reflected in the close values of fracture toughness of 6YSZ, 7YSZ, and 8YSZ ceramics at such same close values of microhardness for these material variants.

Thus, for 6YSZ, 7YSZ, and 8YSZ ceramics, phase balances were defined at which the maximum fracture toughness is reached. The maximum *K*_Ic_ values using both the SENB and Vickers indentation methods for 6YSZ ceramics were obtained at the maximum content of the tetragonal phase (variant 6YSZ-1550), whereas for ceramics with a higher content of the stabilizing Y_2_O_3_ additive (7YSZ and 8YSZ ceramics), the maximum *K*_Ic_ levels were reached at the maximum content of the monoclinic phase (variants 7YSZ-1600 and 8YSZ-1600, respectively).

**Figure 6:**
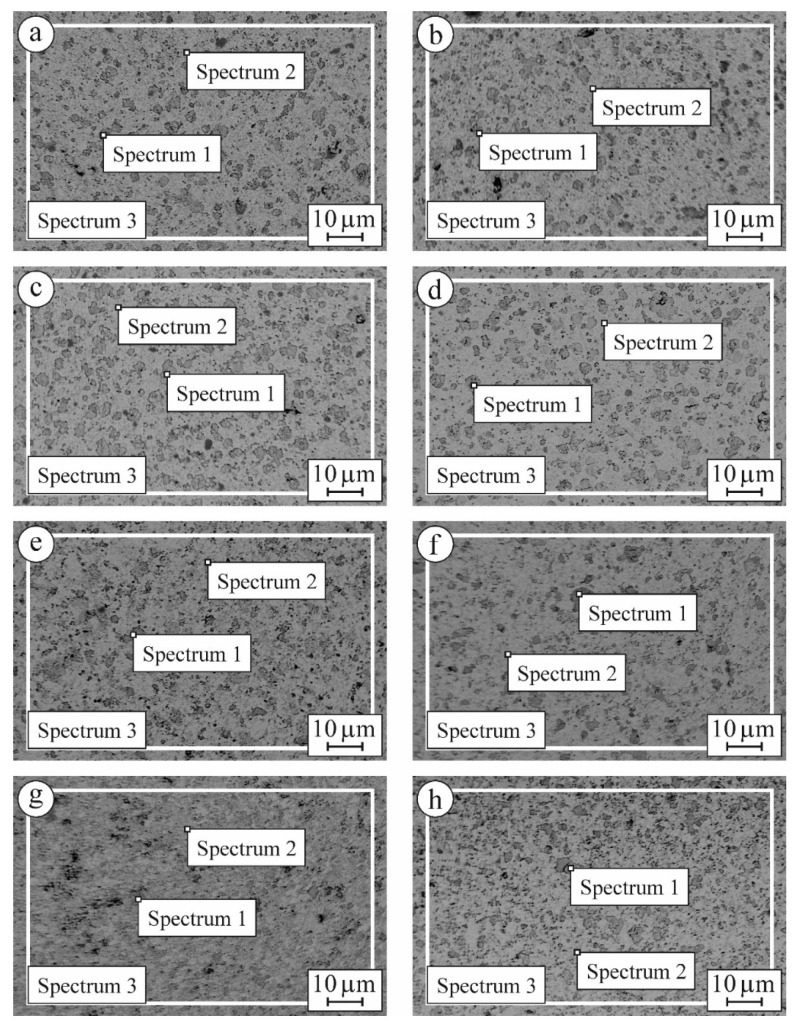
SEM microstructures (BSD images taken at a low magnification) of specimens of (**a**) 6YSZ-1450, (**b**) 6YSZ-1500, (**c**) 6YSZ-1550, (**d**) 6YSZ-1600, (**e**) 7YSZ-1550, (**f**) 7YSZ-1600, (**g**) 8YSZ-1550, and (**h**) 8YSZ-1600 ceramics with corresponding EDX spectra locations (Table 2).

Low-magnification SEM images of the microstructure of the material variants were used for performing local and general EDX analyses (Figure 6). The obtained EDX data sets were ordered in such a fashion (Table 2) that allows for analyzing the availability of the main chemical elements (oxygen, yttrium, and zirconium) in local areas (dark-gray area, spectrum 1; light-gray area, spectrum 2) as well as in general (spectrum 3).

Only variant 7YSZ-1600 exhibits a microstructure which comprises the pure monoclinic phase (see Table 2, spectrum 1 for oxygen, yttrium, and zirconium). A minimum of yttrium percentage is evidence of this assumption. Additionally, we can clearly observe a tendency to increase yttrium content for variants 7YSZ-1550 and 8YSZ-1550, whereas its percentage decreases for variants 6YSZ-1450, 6YSZ-1500, 6YSZ-1550, 6YSZ-1600, and 8YSZ-1600. According to the literature data [19], two mechanisms related to the phenomenon of the yttrium concentration gradient were suggested. One is the concentration gradient-driven mechanism leading to chemical homogenization. The second mechanism relates to the contact point’s geometry which leads to the yttrium diffusion from smaller grains toward larger ones. Therefore, large grains become sufficiently rich in yttrium to form t-ZrO_2_ and c-ZrO_2_ phases while small grains initially rich in yttrium become depleted. Such a phenomenon causes the chemical inhomogeneity of ceramics.

**Figure 7:**
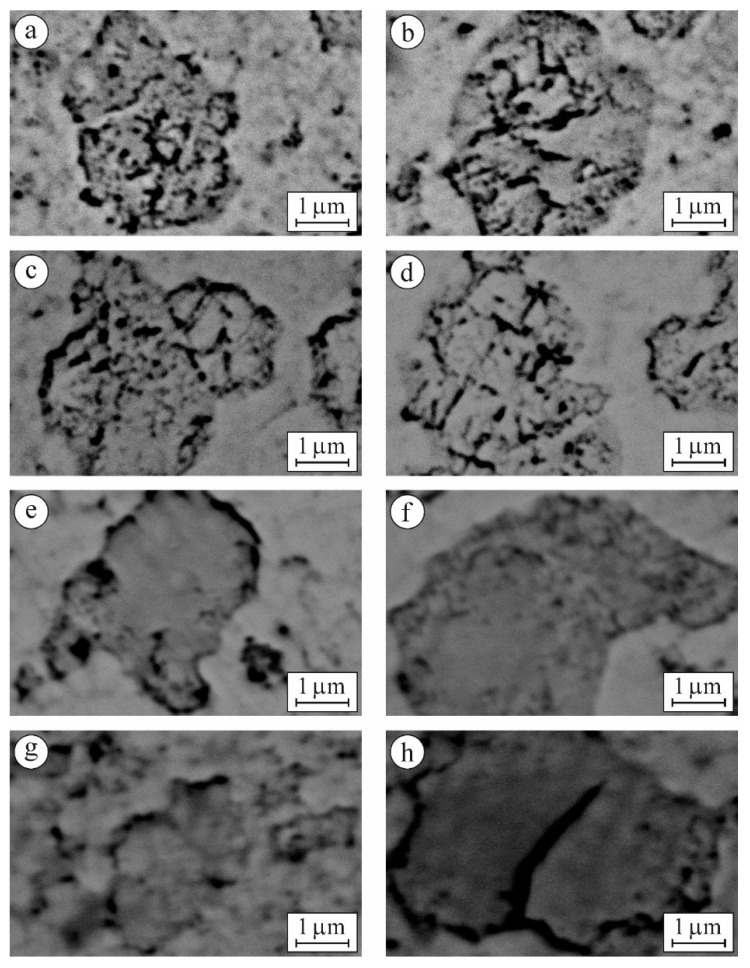
SEM microstructures (BSD images taken at a high magnification) of specimens of (**a**) 6YSZ-1450, (**b**) 6YSZ-1500, (**c**) 6YSZ-1550, (**d**) 6YSZ-1600, (**e**) 7YSZ-1550, (**f**) 7YSZ-1600, (**g**) 8YSZ-1550, and (**h**) 8YSZ-1600 ceramics.

Based on the above assumptions, we were able to identify the m-ZrO_2_ phase areas and analyze their morphology at higher magnification (Figure 7).

**Figure 8:**
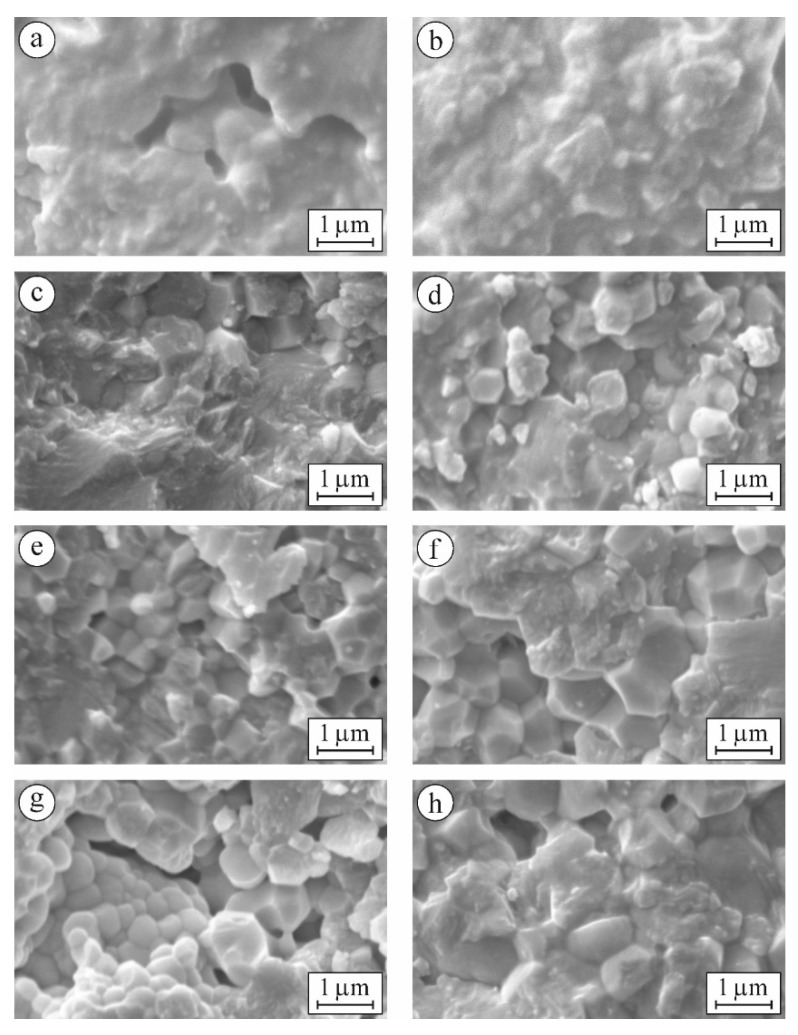
SEM fractography (SE images taken at a high magnification) of specimens of (**a**) 6YSZ-1450, (**b**) 6YSZ-1500, (**c**) 6YSZ-1550, (**d**) 6YSZ-1600, (**e**) 7YSZ-1550, (**f**) 7YSZ-1600, (**g**) 8YSZ-1550, and (**h**) 8YSZ-1600 ceramics.

For variants 6YSZ-1450 and 6YSZ-1500 (Figure 7a,b) there are no signs of any sub-structurization of the m-ZrO_2_ phase agglomerates, whereas for variant 6YSZ-1550 some signs of separate sub-area formation can be observed (Figure 7c), and for variant 6YSZ-1600, clear rectangle-shaped sub-areas can be seen (Figure 7d). Such observations are consistent with peculiarities of fracture surface morphology of the corresponding specimens examined after fracture toughness tests (Figure 8a–d). In particular, the chaotic micro-areas showing fracture mainly along the boundaries of the m-ZrO_2_ phase agglomerates are observed for variants 6YSZ-1450 and 6YSZ-1500 (Figure 8a,b). In contrast, a relief fracture surface can be observed for variant 6YSZ-1550 (Figure 8c) which is evidence of the formation of the microstructure of fully recrystallized m-ZrO_2_ phase grains about 1 μm in size. In this case, both the transgranular fracture of the larger grains and intergranular fracture along distinct boundaries of smaller grains were noted. Additionally, nanoparticles of the t-ZrO_2_ phase about 20–100 nm in size can be clearly seen on the boundaries. Similar to this, a relief fracture surface showing the t-ZrO_2_ phase grains of the increased size (about 150–400 nm) can be observed for variant 6YSZ-1600 (Figure 8d). Such relief fracture patterns for variants 6YSZ-1550 and 6YSZ-1600 are consistent with corresponding average values of both microhardness (Figure 4) and fracture toughness (Figure 5).

For variant 7YSZ–1550 (Figure 7e), separate round-shaped sub-areas about 300–400 nm in size, slightly different in color but without visible boundaries, were detected. It may be suggested that the mentioned sub-areas are yttrium-enriched since maximal yttrium percentage was revealed by EDX analysis in the m-ZrO_2_ phase agglomerates (Table 2). In contrast, in variant 7YSZ-1600, no signs of any sub-area formation of the m-ZrO_2_ phase agglomerates can be seen (Figure 7f). The formation of such fully recrystallized grains of the pure m-ZrO_2_ phase due to minimal yttrium percentage was evidenced by EDX analysis (see Table 2). The morphology of microstructural components was found to be in full accordance with fracture surface patterns of the corresponding specimens (Figure 8e,f). In particular, intergranular fractures along boundaries of the t-ZrO_2_ phase nanoparticles about 200–300 nm in size as well as cleavage facets of the m-ZrO_2_ phase particles can be clearly seen (Figure 8e). For variant 7YSZ-1600, both the transgranular fracture of the larger m-ZrO_2_ phase particles and intergranular fractures along distinct boundaries of the t-ZrO_2_ phase particles about 1 μm in size were noted (Figure 8f). These fracture mechanisms correspond to the highest level of fracture toughness determined by the SENB method (Figure 5b), whereas corresponding average values of microhardness are lower as compared to 6YSZ variants of ceramics (Figure 4).

For variants 8YSZ-1550 and 8YSZ-1600, the average value of microhardness is intermediate among the variants of ceramics under study (Figure 4). Simultaneously, the fracture toughness of both 8YSZ variants determined by the SENB method does not reach high values (Figure 5b). Such behaviors of these material variants can be substantiated taking into account the peculiarities of the morphology of microstructural components (Figure 7g,h) and the corresponding fracture micromechanisms (Figure 8g,h). In particular, from the point of view of yttrium distribution between the t-ZrO_2_ and m-ZrO_2_ phases (Table 2), a steep decrease in yttrium percentage was observed for the m-ZrO_2_ phase agglomerates with a sintering temperature increase. However, this did not allow the (t-m) ZrO_2_ phase transformation [58] to occur to a great extent and thus did not provide high values of fracture toughness even for variant 8YSZ-1600. The morphology of the m-ZrO_2_ phase agglomerates was characterized by blurred contours and the t-ZrO_2_ phase particles were quite distinct for variant 8YSZ-1550 (Figure 7g). In contrast, greatly disintegrated m-ZrO_2_ phase agglomerates were seen in the microstructure of variant 8YSZ-1600 (Figure 7h). The corresponding fracture mechanism in a specimen of variant 8YSZ-1550 was intergranular due to relatively large pores (Figure 8g), whereas in a specimen of variant 8YSZ-1600, it comprised both transgranular and intergranular fracture microregions (Figure 8h).

Thus, based on the results of the microhardness test and fracture toughness test using the SENB method, it can be concluded that the best variant of material is that of 7YSZ ceramics sintered at a temperature of 1600 °C (variant 7YSZ-1600).

## 4. Conclusions

1. In this work, the conditions for the formation of tetragonal, monoclinic, and cubic phases of zirconia in 6YSZ, 7YSZ, and 8YSZ ceramics have been substantiated.
2. The dependences of phase composition and mechanical properties of the studied YSZ ceramics on the sintering temperature have been analyzed.
3. The fracture toughness of YSZ ceramics is related to the phase transformations occurring in the material in the presence of a stabilizing additive. By comparing the mechanical behaviors of studied material variants, it was found that 7YSZ ceramics sintered at 1600 °C have the highest level of fracture toughness.

## Figures and Tables

**Table 1:** Chemical composition and sintering modes of variants of the investigated material.

Variant	Content of Y_2_O_3_, mol%	Sintering Mode	
		Temperature, °C	Time, h
6YSZ–1450	6	1450	2
6YSZ–1500	6	1500	2
6YSZ–1550	6	1550	2
6YSZ–1600	6	1600	2
7YSZ–1550	7	1550	2
7YSZ–1600	7	1600	2
8YSZ–1550	8	1550	2
8YSZ–1600	8	1600	2

**Table 2:** The percentage (wt%) of the main chemical elements (oxygen, yttrium, and zirconium) present in local areas (spectrums 1 and 2) and in general (spectrum 3) according to the local and general EDX analyses of the investigated material variants (see Figure 6).

Chemical Element	Spectrum	Variants of Material							
		6YSZ-1450	6YSZ-1500	6YSZ-1550	6YSZ-1600	7YSZ-1550	7YSZ-1600	8YSZ-1550	8YSZ-1600
	1	14.4	14.88	13.87	14.93	6.93	17.82	10.39	16.41
O	2	15.16	17.31	15.59	14.82	17.6	17.06	16.78	17.44
	3	15.09	15.73	15.05	15.06	15.84	17.31	16.01	16.75
	1	5.98	5.99	6.99	9.1	19.48	3.68	15.42	6.11
Y	2	2.26	0.59	2.26	1.32	0	0.91	0.38	1.25
	3	3.48	2.92	2.8	2.79	3.26	1.49	3.2	2.68
	1	79.62	79.13	79.14	75.97	73.59	78.5	74.19	77.48
Zr	2	82.58	82.1	82.15	83.86	82.4	82.03	82.84	81.31
	3	81.43	81.35	82.15	82.15	80.9	81.2	80.79	80.57

## Data Availability

All the supporting and actual data are presented in the manuscript.
